# Crystalliferous *Bacillus cereus* group bacteria from a Maryland hardwood forest are dominated by psychrotolerant strains

**DOI:** 10.1002/mbo3.189

**Published:** 2014-07-01

**Authors:** Michael B Blackburn, Phyllis A W Martin, Daniel Kuhar, Robert R Farrar, Dawn E Gundersen-Rindal

**Affiliations:** 1 Invasive Insect Biocontrol and Behavior Laboratory, USDA/ARSBeltsville, Maryland, 20705

**Keywords:** *Bacillus cereus*, *Bacillus thuringiensis*, *Bacillus weihenstephanensis*, multilocus sequence analysis, psychrotolerance

## Abstract

Crystal-forming bacteria of the *Bacillus cereus* group were isolated from soil samples collected at different elevations within a mixed hardwood forest in central Maryland, and their phylogenetic relationships determined by multilocus sequence analysis. The vast majority of isolates obtained were associated with two phylogenetic groups known to be psychrotolerant, with very few isolates representing phylogenetic groups more typically associated with *Bacillus thuringiensis*. Isolates from the psychrotolerant groups were found to grow on solid media at 7°C. Isolates of 11 highly related, novel sequence types (STs) from the psychrotolerant group that includes *Bacillus weihenstephanensis* were generally found at higher elevations, and were not associated with soils near streams. Isolates of two related STs from the second psychrotolerant group were nearly always found at the bottoms of ravines near streams, in areas abundant in earthworm castings.

## Introduction

The *Bacillus cereus* group consists of seven closely related species: *Bacillus cereus*, *Bacillus anthracis*, *Bacillus thuringiensis* (*Bt*), *Bacillus mycoides*, *Bacillus pseudomycoides*, *Bacillus weihenstephanensis*, and *Bacillus cytotoxicus*. The sister species are distinguished from *B. cereus* largely on the basis of a few dominant phenotypic features. At the phylogenetic level, however, the species generally occupy overlapping ranges with indistinct boundaries (Helgason et al. 2004; Priest et al. 2004; Guinebretière et al. 2008; Didelot et al. 2009; Tourasse et al. 2011).

Widely distributed in the environment, members of the *B. cereus* group are capable of occupying a wide variety of environmental niches. Recently, Guinebretière et al. (2008) identified seven phylogenetically distinct groups (I–VII) within the *B. cereus* sensu lato, and demonstrated that these differed in their ability to grow at different temperatures. Phenotypically, the entire group was bracketed by the psychrotolerant *B. weihenstephanensis* and *B. mycoides* of Group VI, and the thermotolerant *B. cytotoxicus* (Guinebretière et al. 2013) of Group VII. While Group VI strains were capable of growth between 7 and 37°C, Group VII exhibited growth between 20 and 50°C. For both the psychrotolerant *B. weihenstephanensis* and the thermotolerant *B. cytotoxicus*, growth at low and high temperatures, respectively, are key features in differentiating these species from other members of the *B. cereus* group (Lechner et al. 1998; Guinebretière et al. 2013) and may contribute to their considerable potential as food-borne pathogens (Thorsen et al. 2006; Contzen et al. 2014).

*Bacillus thuringiensis*, differentiated from *B. cereus* by its production of parasporal protein crystals that are often insecticidal, is widely used in agriculture for insect suppression. Although the prior phylogenetic studies cited above have shown that many *Bts* tend to cluster in a particular group (Group IV of Guinebretière et al. 2008), they can be found interspersed in other groups as well. Recently, a number of reports have emerged describing crystal-forming strains of the *B. cereus* group that are also psychrotolerant, and or phylogenetically related to *B. weihenstephanensis* (Guinebretière et al. 2008; Bartoszewicz et al. 2009; Soufiane and Côté 2010; Drewnowska and Święcicka 2013; Święcicka et al. 2013).

We have recently examined the spatial and phylogenetic distribution of crystal-forming soil bacteria in the *B. cereus* group from a mature mixed hardwood forest in Maryland. Surprisingly, the local population of crystal formers belonged almost exclusively to two psychrotolerant groups described by Guinebretière et al. (2008). Representatives of the two groups were found to occur in distinct soil habitats.

## Materials and Methods

### Sampling

Soil samples were collected in early September 2009 from a mature hardwood forest in central Maryland. Elevations in the sampling region ranged from ca. 135 to 155 m above sea level. Weather records from 1993 to the present for a weather station 4.5-km east of the sampled area indicate mean monthly temperatures of −0.3°C for January and 23.5°C for July (National Climate Data Center, http://ncdc.noaa.gov/land-based-station-data/land-based-datasets/climate-normals). Temperatures at the time of sampling were very warm, with cursory measurements indicating soil surface temperatures of 19–20°C. At higher elevations, the forest was composed primarily of mixed oak (*Quercus* spp.), American beech (*Fagus grandifolia*), Red maple (*Acer rubrum*), and occasional Virginia pine (*Pinus virginiana*); at lower elevations the dominant species was Tulip tree (*Liriodendron tulipifera*). Sampling was conducted along transects oriented perpendicularly across ravines so as to include both slopes; transect lengths were scaled according to the dimensions of the ravine and ranged from 110 to 240 m. Three soil samples were collected 3 m apart near the top, middle, and bottom of each slope, resulting in nine samples per slope and 18 samples per transect. After loose leaf litter was brushed away, samples were scooped from the surface 2–3 cm of soil using sterile 50 mL centrifuge tubes. A total of 90 samples were collected in this way from five transects. Four of the ravines chosen for study were associated with permanent streams; two of these had U-shaped profiles with distinct floodplains, and two were V-shaped. The fifth ravine was V-shaped and associated with an ephemeral stream.

### Isolation of bacteria

To compensate for differences in moisture content, samples were allowed to dry at ambient room temperature. For each sample, 100 mg of dry soil was added to 10 mL of sterile deionized water in a 15-mL centrifuge tube. The tube was then vortexed for 1 min. One hundred microliters of the resulting suspension was then pasteurized for 3 min at 80°C. Pasteurized suspensions were then diluted 1:10 in sterile deionized water. Thirty microliters of the diluted suspension was then plated on T3 agar (Travers et al. 1987) in each of three 100 mm Petri dishes. Plates were incubated at 24°C for 96 h.

Starting from a randomly selected point on a plate and working clockwise, the first 30 colonies from each sample were examined microscopically for the presence of parasporal bodies. Each colony in which parasporal bodies were found was then streaked on T3 agar in a 50-mm Petri dish, and subcultured until pure cultures were obtained.

### Multilocus sequence analysis

Isolates were phylogenetically characterized using the multilocus sequence analysis (MLSA) scheme of Priest et al. (2004). Sequences for the multiple loci were amplified for each isolate using primers for the *glpF*, *gmk*, *ilvD*, *pta*, *pur*, *pycA*, and *tpi* loci described in that earlier study, with the exception of a *pta* forward primer (ptaF1 5′-GCGTTTAGCAAAAGAAGAGTTAGTA-3′) Blackburn et al. (2011). For PCR, 35 cycles were conducted in a model 9700 thermocycler (Applied Biosystems, Foster City, CA) using 30 sec denaturation at 94°C, 1.5-min annealing at 55°C, and 2-min primer extension (10-min in final cycle) at 72°C. Each gene amplicon was sequenced directly. Products were separated on 1.5% NuSieve agarose gel (FMC, Rockland, ME) in modified 1× TAE (0.04 mol/L Tris-acetate and 0.1 mmol/L ethylenediaminetetraacetic acid), and excised for sequencing using ABI BigDye 3.1 (Applied Biosystems), using the amplification primers. Cycle sequencing conditions were 35 cycles at 96°C, 10 sec; 50°C, 5 sec; 60°C for 4 min. Automatic sequencing was carried out on an ABI Prism Model 3130xl (Applied Biosystems). Sequences were edited and assembled using the SeqMan component of DNASTAR (DNASTAR, Inc., Madison, WI). Sequence types (STs) were determined by BLAST searches (Altschul et al. 1990) of the PubMLST database for the *B. cereus* group (Jolley et al. 2004). Novel allele sequences and allelic profiles were deposited in the database (http://pubmlst.org/bcereus/) and STs assigned. Phylogenetic analysis was conducted with MEGA 5 software (Tamura et al. 2011) using the maximum likelihood method. The analysis was performed on concatenated sequences for all seven loci from STs found in the current study, and all additional STs in the PubMLST database at the time (ST-1 through ST-610). Assignment of STs into phylogenetic groups described by Guinebretière et al. (2008) was accomplished using University of Oslo's *B. cereus* group MultiLocus and MultiData Typing website (http://mlstoslo.uio.no) using HyperCAT (Tourasse et al. 2010).

### Toxicity screening and low-temperature growth

Eggs of the gypsy moth, *Lymantria dispar* (L.) (Lepidoptera: Erebidae) were obtained from USDA/APHIS, Otis ANGB, MA. Larvae were reared to the second instar on artificial diet (Bell et al. 1981). Freeze-dried pellets of artificial diet were placed in plastic bioassay trays (Bio-BA 128^©^; BioServ, Frenchtown, NJ) and rehydrated with whole cultures of bacteria (Martin 2004). Four pellets were treated with each bacterial isolate. Two larvae were placed on each pellet, for a total of eight larvae per isolate. Up to 37 isolates were tested on each of four dates, plus a positive control of IBL455 (*B. thuringiensis* subsp. *kurstaki*, originally isolated from Dipel®, Abbott Laboratories, Chicago, IL), and a negative control of water only, on each date. Each isolate was tested only once. Larvae were held at 27°C and scored (alive or dead) at 3 and 6 days. All isolates were tested for the ability to grow from spores and vegetative cells at 7°C on Luria agar.

## Results and Discussion

**Figure 1:**
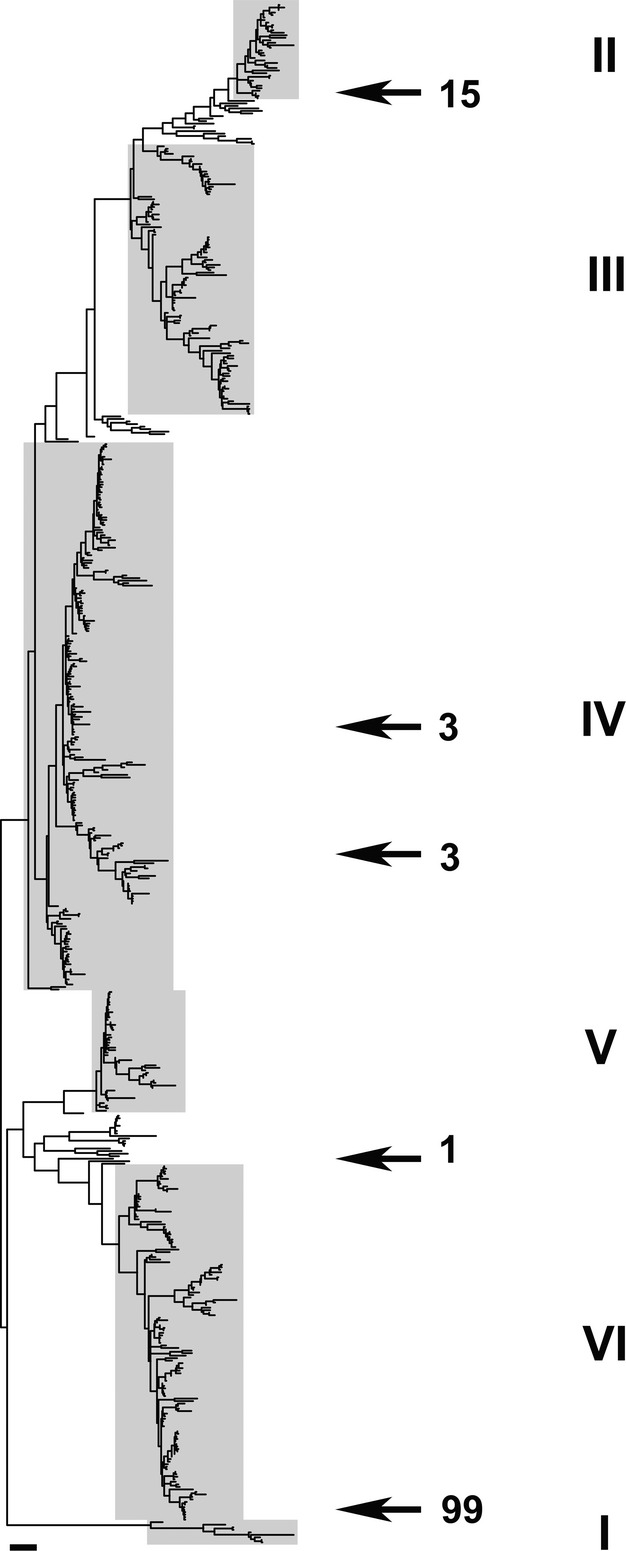
Multilocus sequence analysis phylogeny of *Bacillus cereus* sequence types 1–610 by maximum likelihood. Roman numerals corresponding to shaded regions refer to the groups identified by Guinebretière et al. (2008). Numbers with arrows indicate the number of recovered isolates from that phylogenetic position.

From 90 soil samples we obtained 121 isolates that produced parasporal crystals. The distribution of isolates and STs among transects was variable. Some areas produced abundant isolates from multiple samples while other areas produced none, despite there being no obvious environmental differences. The number of samples producing isolates from the top, middle, and bottom of slopes was nearly identical (15, 14, and 13, respectively); however, a significantly greater number of isolates were recovered from top to middle slope samples compared with bottom samples; while top and middle slope samples yielded 50 and 51 isolates, respectively, bottom samples yielded only 20 (*χ*^2^ = 15.00; *P *=* *0.0010). Among the isolates were representatives of 17 STs, 16 of which were novel (Table1). Phylogenetic analysis of the novel STs based on concatenated allele sequences (Fig.1) revealed that the vast majority were associated with Groups VI and II, which represent the two currently recognized groups of psychrotolerant strains (Guinebretière et al. 2008).

**Table 1:** Characteristics and distribution of sequence types.

Group^1^	ST	Crystal type	Isolates	Samples with ST	Transects with ST	Top of slope	Middle of slope	Bottom of slope	Growth at 7°C
IV	8	bp	3	3	1	1		2^2^	N
II	551	trap	9	6	2		1	8	Y
IV	596	sm bp	3	1	1	3			N
II	597	bp	1	1	1	1			Y
VI	598	bp	4	2	1	4			Y
VI	599	bp/cu	4	4	2		3	1^2^	Y
II	600	am/cu	5	2	1			5	Y
VI	601	att irr	10	8	3	5	2	3^2^	Y
VI	602	bp	3	3	1	2	1		Y
VI	603	bp/cu	39	9	2	3	35	1^2^	Y
VI	604	att irr	1	1	1		1		Y
VI	605	att cu	25	11	4	20	5		Y
VI	606	bp	10	1	1	10			Y
VI	607	bp	1	1	1	1			Y
VI	608	att am	1	1	1		1		Y
VI	609	att am	1	1	1		1		Y
	610	bp	1	1	1		1		Y

Am, amorphous; att, attached; bp, bipyramidal; cu, cubic; irr, irregular; sm, small; trap, trapezoidal; ST, sequence type.

1 Refers to the group designation of Guinebretière et al. (2008).

1 Isolates indicated were recovered from the bottom of the dry transect (ephemeral stream).

**Figure 2:**
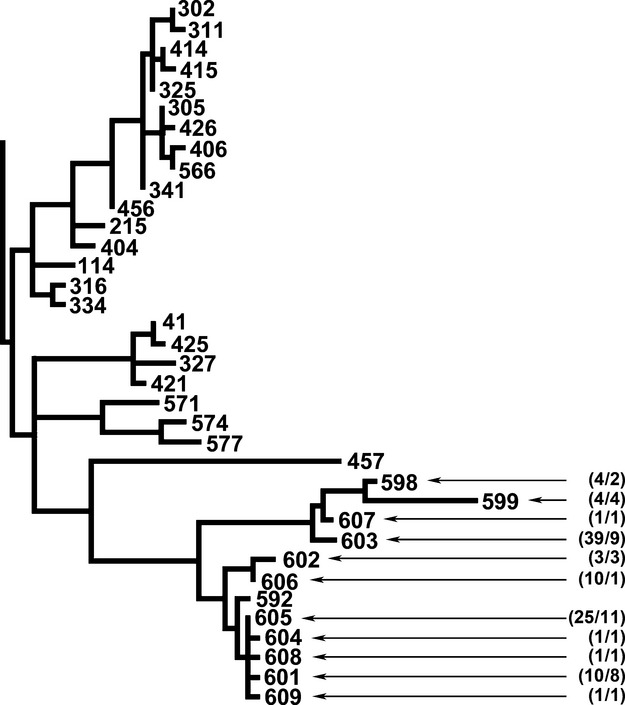
Detail from the tree presented in Figure1 showing the phylogenetic relationships between Group VI sequence types (STs) from this study and their near neighbors. Numbers in parentheses indicate the number of isolates found/number of soil samples containing the indicated STs.

Group VI comprises primarily *B. weihenstephanensis*, *B. mycoides*, and *B. cereus* that were identified prior to the description of *B. weihenstephanensis*. In recent years a growing number of reports have appeared in the literature describing crystal-forming strains belonging to this group, including *Bt* serovars *navarrensis*, *bolivia*, and *vazensis* (Guinebretière et al. 2008; Bartoszewicz et al. 2009; Soufiane and Côté 2010; Drewnowska and Święcicka 2013; Święcicka et al. 2013). Group VI isolates were by far the most abundant in our samples, accounting for 99 isolates in 11 STs. Another ST in this cluster, ST-592, was identified in a preliminary survey of the area. As can be seen in Figure2, the Group VI STs identified from this region were highly related, and did not bracket other STs in the PubMLST database. Sequence type 605 was the most widespread, being isolated from 11 samples across four of the transects. Members of Group VI were absent from bottom sampling elevations in ravines with permanent streams; only five isolates were found in three adjacent bottom samples from the ravine with an ephemeral stream (which was dry at the time of sampling). Sequence type-601, ST-604, ST-605, ST-608, and ST-609 shared the common feature of having crystals that remained attached to the spore, although the morphology of the crystal appeared to vary between irregular, amorphous, and cubic. Sequence type-601, ST-604, ST-608, and ST-609 each differed from ST-605 by single-nucleotide substitutions. The remaining STs from this group (ST-598, ST-599, ST-602, ST-603, ST-606, and ST-607) produced bipyramidal crystals that detached from the spore. All isolates belonging to Group VI grew on L agar at 7°C. A single isolate that appeared among unclustered STs near Group VI was designated ST-610. The ST-610 *gmk* and *ilvD* allele sequences deviated from their nearest neighbors in the PubMLST database by 15 and 21 nucleotides, respectively, and for all alleles by a sum of 59 substitutions. The ST-610 isolate produced bipyramidal crystals and also grew at 7°C.

**Figure 3:**
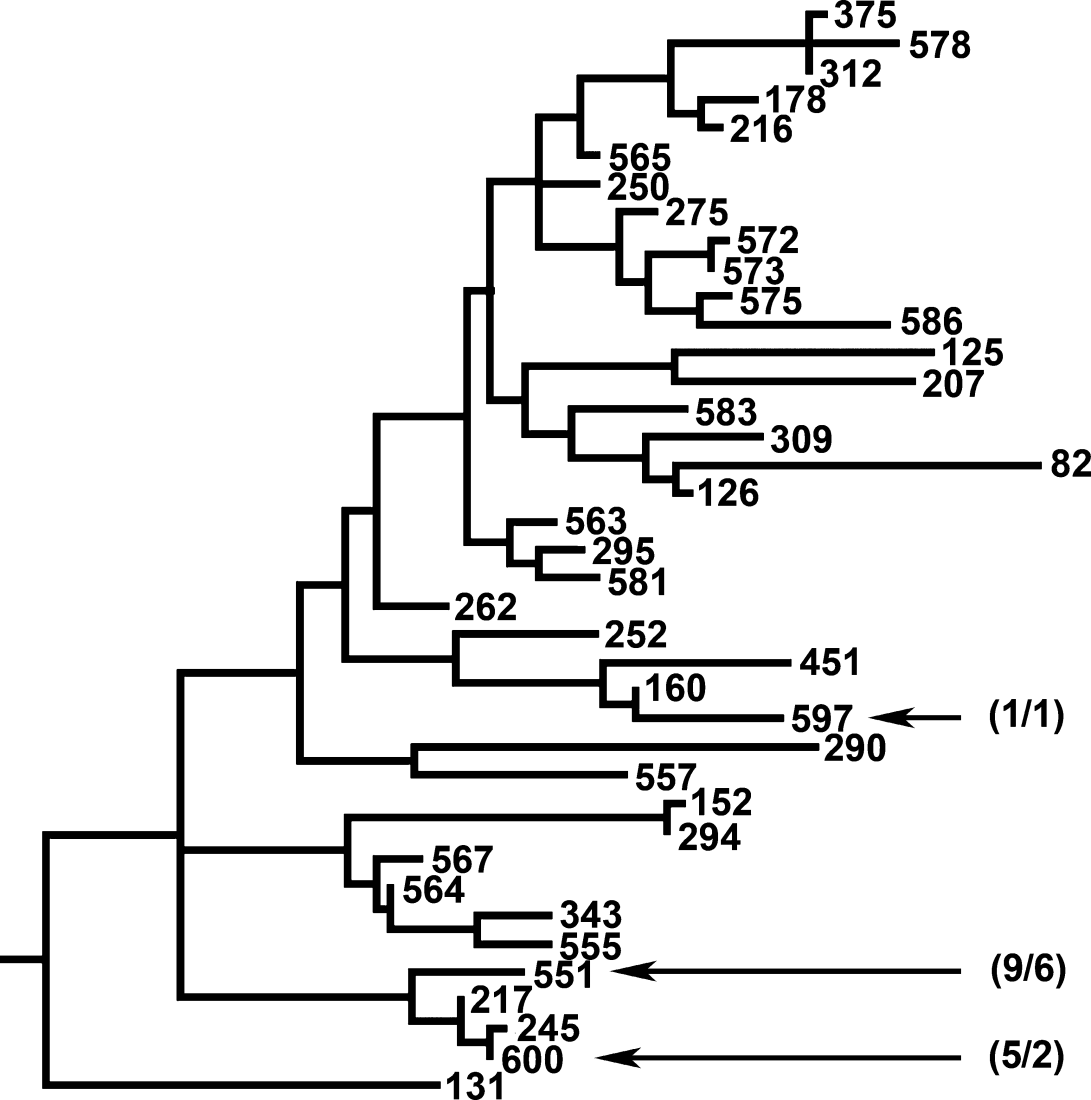
Detail from the tree presented in Figure1 showing the phylogenetic relationships between Group II sequence types (STs) from this study and their near neighbors. Numbers in parentheses indicate the number of isolates found/number of soil samples containing the indicated STs.

Most of the remaining isolates from our samples belonged to Group II and were, like the Group VI isolates, highly related to each other (Fig.3). Sequence type-551 was found almost exclusively in “bottom” samples from the two U-profile ravines with distinct floodplains. The surface soil in these locations at the time of sampling was free of leaf litter and composed largely of earthworm castings, leading us to suspect that the lifecycle of these bacteria is linked in some way to earthworms. Sequence type-551 produces unusually shaped crystals that appear to be trapezoidal with rounded corners. Closely related to ST-551, ST-600 was isolated from two samples at the bottom of a V-shaped ravine with a permanent stream. Crystals produced by ST-600 were amorphous to cubic in shape. A single isolate of ST-597, which produced bipyramidal crystals, also clustered with Group II. Unlike ST-551 and ST-600 isolates, the ST-597 isolate was recovered from a slope top sample. Like the Group VI isolates, Group II isolates grew at 7°C. A number of *Bt* serotype strains were reported to occur in Group II, including *toguchini*, *andaluciensis*, *balerica*, *muju*, *argentinensis*, *sylvestriensis*, and *sinensis* (Guinebretière et al. 2008).

The remaining isolates were associated with Group IV and included three representatives of ST-8, which includes *Bt kurstaki*, and three isolates of ST-596. Sequence type-596 appears closely related to ST-240 which corresponds to *Bt toumanoffi*. The original isolate representing ST-240 was a *Bt toumanoffi* (Terrence Leighton and Katie Wheeler, unpubl. data) and ST-240 strain IBL 200 has a predicted flagellar protein sequence (GenBank accession # EEM97050) identical to that reported for *Bt toumanoffi* (Xu and Côté 2008; GenBank accession # ABD33729.2).

Aside from the three ST-8 isolates, which killed all gypsy moth larvae within 3 days, preliminary screening of isolates revealed variable levels of toxicity, even within STs. For instance, groups of larvae fed the 39 isolates of ST-603 displayed 6-day mortalities ranging from 0% to 100%, with a mean (±SE) of 49 ± 4.3%. Twenty-five isolates of another common Group VI representative, ST-605 produced 6 day mortality of 46 ± 6.1%. Ten isolates of the most common Group II member, ST-551, resulted in 35 ± 7.1% mortality. No control mortality was observed in any of the assays. Assay results of all individual isolates are presented in Table S1.

Browsing the PubMLST database reveals that the more abundant *Bt* STs, such as ST-8, ST-16, ST-23, and ST-171, appear to have nearly global distributions. An MLSA survey of isolates from a worldwide *Bt* collection in our laboratory (Blackburn et al. 2013) revealed only one isolate each of STs belonging to Groups VI and II (ST-592 and ST-551, respectively) and these were isolated from the same forest sampled in the present study. The abundance of psychrotolerant isolates with previously undescribed STs found in this study, and the paucity of more commonly encountered phylogenetic groups associated with *Bt* was unexpected. However, two other surveys of the *B. cereus* group from forest soils have revealed some parallels with the current study. In a survey of forest soils in Versailles, France, Sorokin et al. (2006) also found psychrotolerant Group VI isolates in unexpected abundance, although these did not produce crystals. In contrast to our results, however, that survey revealed numerous *Bt* isolates belonging to Group IV, with clear evidence of the Kurstaki, Tolworthi, and Sotto lineages described earlier by Priest et al. (2004). More recently, in a phylogenetic survey of the *B. cereus* group in northeastern Poland, Drewnowska and Święcicka (2013) found that isolates from an old growth forest were primarily from Group VI, and the majority of these formed parasporal inclusions. As with our survey, very few isolates from forest soil were associated with Group IV, however, among crystal-forming isolates collected from a farm and marshland in adjacent regions proportionally fewer isolates were associated with Group VI and more were associated with Group IV. Thus, the studies that have specifically surveyed forest soils have reported a higher proportion of Group VI isolates, which may or may not produce parasporal crystals. Whether this is due to cooler soil temperatures in forests or some other factor will require further investigation. In a study comparing the relative distributions of psychrotolerant and mesophilic strains in tropical, temperate, and alpine environments, it was found that psychrotolerant strains were absent from tropical soils, predominated in alpine soils, and were present in approximately equal abundance with mesophilic strains in temperate soils (von Stetten et al. 1999).

The tight phylogenetic clustering of isolates found in this study, particularly the Group VI isolates, suggests that the members of this group have either maintained their association with each other over time, or have rapidly evolved at their current location, possibly displacing the more widely encountered STs (Blackburn et al. 2013). Considering the ability of these bacteria to propagate at temperatures well below those required for replication of Group IV *Bts*, the latter may be plausible. Many of our samples contained more than one ST from the Group VI, and a single slope of one transect produced representatives of ST-8, ST-598, ST-599, ST-601, ST-603, ST-605, ST-608, and ST-609, with two adjacent samples containing four STs and two others with three STs. Thus, it does not appear that the presence of a particular ST excludes others from that location, and they may actually be clustered in locations favorable for their growth, although this will require a much more extensive sampling to determine.

The data from this preliminary study indicate that the distribution of these bacteria is nonuniform across habitat that appears superficially uniform. We do not yet know if the distribution is relatively static or dynamic, but the ability to identify population sources and sinks may allow identification of environmental factors that are correlated with population density.

## Supporting Information

Table S1 Crystal-forming *Bacillus* isolates recovered, their sequence types, toxicity, and distribution.
