# Supplementary material for: Crystalliferous Bacillus cereus group bacteria from a Maryland hardwood forest are dominated by psychrotolerant strains
Source: Microbiologyopen. 2014 Jul 1;3(4):578–84. doi: 10.1002/mbo3.189 (PMC4287184; doi:10.1002/mbo3.189)
Supplement: Table S1 — Crystal-forming Bacillus isolates recovered, their sequence types, toxicity, and distribution. [file mbo30003-0578-sd1.pdf]

Supplemental Table 1
Crystal forming Bacillus isolates recovered, their sequence types, toxicity and distribution.

Isolate	Sequence Type	Group	Crystal morph.	3 day gypsy moth mort. dead/tested	6 day gypsy moth mort. dead/tested	Transect/Slope	Position on slope
IBL3757	8	IV	bp cu	8/8	8/8	3/a	top
IBL3777	8	IV	bp	8/8	8/8	3/a	bottom
IBL3782	8	IV	bp cu	8/8	8/8	3/b	bottom
IBL3680	551	II	trap	3/8	3/8	1/a	bottom
IBL3681	551	II	trap	0/8	1/8	1/b	bottom
IBL3682	551	II	trap	0/8	0/8	1/b	bottom
IBL3683	551	II	trap	0/8	1/8	1/b	bottom
IBL3791	551	II	trap	1/8	3/8	4/a	bottom
IBL3794	551	II	trap	0/8	5/8	4/a	bottom
IBL3792	551	II	trap	3/8	4/8	4/b	bottom
IBL3793	551	II	trap	2/8	3/8	4/b	bottom
IBL3795	551	II	trap	3/8	5/8	4/b	middle
IBL3788	596	IV	sm bp	3/8	4/8	4/a	top
IBL3789	596	IV	sm bp	2/8	8/8	4/a	top
IBL3790	596	IV	sm bp	3/8	5/8	4/a	top
IBL3720	597	II	bp	0/8	3/8	2/b	top
IBL3721	598	VI	bp	0/8	0/8	2/b	top
IBL3722	598	VI	bp	0/8	1/8	2/b	top
IBL3723	598	VI	bp	1/8	3/8	2/b	top
IBL3727	598	VI	bp	7/8	8/8	3/a	top
IBL3747	599	VI	bp cu	2/8	4/8	3/a	middle
IBL3763	599	VI	bp cu	0/8	3/8	3/a	middle
IBL3778	599	VI	bp	1/8	6/8	3/a	bottom
IBL3803	599	VI	bp	1/8	3/8	5/b	middle
IBL3796	600	II	cu	4/8	5/8	5/a	bottom
IBL3797	600	II	am cu	3/8	5/8	5/a	bottom
IBL3798	600	II	am	0/8	2/8	5/a	bottom
IBL3799	600	II	am	3/8	4/8	5/a	bottom
IBL3800	600	II	am	5/8	7/8	5/b	bottom
IBL3689	601	VI	att cu	0/8	0/8	1/b	top
IBL3719	601	VI	att cu	2/8	3/8	2/b	top
IBL3725	601	VI	att ir	0/8	8/8	3/a	top
IBL3729	601	VI	att cu	0/8	1/8	3/a	top
IBL3760	601	VI	att irr	0/8	0/8	3/a	top
IBL3776	601	VI	att irr	3/8	5/8	3/a	bottom
IBL3779	601	VI	att irr	2/8	2/8	3/a	bottom
IBL3780	601	VI	att irr	0/8	3/8	3/a	middle

IBL3781	601	VI	att irr	5/8	6/8	3/a	bottom
IBL3785	601	VI	att irr	1/8	1/8	3/b	middle
IBL3676	602	VI	bp	0/8	2/8	1/a	top
IBL3677	602	VI	bp	0/8	0/8	1/a	top
IBL3678	602	VI	bp	3/8	4/8	1/a	middle
IBL3718	603	VI	bp	1/8	2/8	2/b	top
IBL3730	603	VI	bp cu	0/8	7/8	3/a	top
IBL3732	603	VI	bp cu	4/8	7/8	3/a	middle
IBL3733	603	VI	bp cu	0/8	0/8	3/a	middle
IBL3734	603	VI	bp cu	0/8	5/8	3/a	middle
IBL3735	603	VI	bp cu	0/8	4/8	3/a	middle
IBL3736	603	VI	bp cu	1/8	4/8	3/a	middle
IBL3737	603	VI	bp cu	1/8	1/8	3/a	middle
IBL3739	603	VI	bp cu	0/8	0/8	3/a	middle
IBL3740	603	VI	bp cu	0/8	3/8	3/a	middle
IBL3741	603	VI	bp cu	1/8	1/8	3/a	middle
IBL3743	603	VI	bp cu	0/8	0/8	3/a	middle
IBL3744	603	VI	bp cu	0/8	3/8	3/a	middle
IBL3745	603	VI	bp cu	1/8	2/8	3/a	middle
IBL3746	603	VI	bp cu	2/8	6/8	3/a	middle
IBL3748	603	VI	bp cu	2/8	7/8	3/a	middle
IBL3749	603	VI	bp cu	0/8	3/8	3/a	middle
IBL3750	603	VI	bp cu	0/8	6/8	3/a	middle
IBL3752	603	VI	bp cu	0/8	2/8	3/a	middle
IBL3753	603	VI	bp cu	1/8	4/8	3/a	middle
IBL3754	603	VI	bp cu	0/8	5/8	3/a	middle
IBL3755	603	VI	bp cu	1/8	3/8	3/a	middle
IBL3756	603	VI	bp cu	0/8	5/8	3/a	middle
IBL3758	603	VI	bp cu	0/8	4/8	3/a	top
IBL3762	603	VI	bp cu	0/8	3/8	3/a	middle
IBL3764	603	VI	bp cu	0/8	3/8	3/a	middle
IBL3765	603	VI	bp cu	3/8	4/8	3/a	middle
IBL3767	603	VI	bp cu	0/8	3/8	3/a	bottom
IBL3768	603	VI	bp cu	5/8	7/8	3/a	middle
IBL3769	603	VI	bp cu	3/8	3/8	3/a	middle
IBL3770	603	VI	bp cu	7/8	8/8	3/a	middle
IBL3771	603	VI	bp cu	1/8	2/8	3/a	middle
IBL3772	603	VI	bp cu	4/8	4/8	3/a	middle
IBL3773	603	VI	bp cu	4/8	5/8	3/a	middle
IBL3774	603	VI	bp cu	5/8	5/8	3/a	middle
IBL3775	603	VI	bp cu	3/8	6/8	3/a	middle
IBL3784	603	VI	bp cu	3/8	7/8	3/b	middle
IBL3786	603	VI	bp cu	1/8	6/8	3/b	middle

IBL3787	603	VI	bp cu	1/8	2/8	3/b	middle
IBL3685	604	VI	att irr	0/8	1/8	1/a	middle
IBL3684	605	VI	att cu	0/8	0/8	1/a	middle
IBL3686	605	VI	att irr	2/8	4/8	1/a	middle
IBL3687	605	VI	att cu	4/8	4/8	1/b	top
IBL3688	605	VI	att cu	1/8	1/8	1/b	top
IBL3690	605	VI	att cu	0/8	5/8	2/a	top
IBL3691	605	VI	att cu	0/8	1/8	2/a	top
IBL3692	605	VI	att cu	0/8	3/8	2/a	top
IBL3694	605	VI	att cu	2/8	2/8	2/a	top
IBL3700	605	VI	att cu	1/8	3/8	2/a	top
IBL3708	605	VI	att cu	0/8	1/8	2/a	top
IBL3709	605	VI	att cu	2/8	7/8	2/a	top
IBL3712	605	VI	att cu	3/8	8/8	2/a	top
IBL3713	605	VI	att cu	1/8	5/8	2/a	top
IBL3714	605	VI	att cu	4/8	7/8	2/a	top
IBL3715	605	VI	att cu	2/8	8/8	2/a	top
IBL3716	605	VI	att cu	0/8	4/8	2/a	top
IBL3717	605	VI	att cu	1/8	4/8	2/a	middle
IBL3724	605	VI	att cu	0/8	3/8	3/a	top
IBL3726	605	VI	att cu	2/8	7/8	3/a	top
IBL3728	605	VI	att cu	0/8	4/8	3/a	top
IBL3731	605	VI	att cu	0/8	4/8	3/a	top
IBL3759	605	VI	att cu	0/8	1/8	3/a	top
IBL3761	605	VI	att cu	0/8	0/8	3/a	top
IBL3801	605	VI	att cu	2/8	5/8	5/b	middle
IBL3802	605	VI	att cu	0/8	1/8	5/b	middle
IBL3695	606	VI	bp	1/8	2/8	2/a	top
IBL3696	606	VI	bp cu	1/8	3/8	2/a	top
IBL3697	606	VI	bp	0/8	8/8	2/a	top
IBL3698	606	VI	bp	2/8	2/8	2/a	top
IBL3699	606	VI	bp	1/8	2/8	2/a	top
IBL3702	606	VI	bp	0/8	5/8	2/a	top
IBL3704	606	VI	bp	2/8	2/8	2/a	top
IBL3705	606	VI	bp	0/8	3/8	2/a	top
IBL3706	606	VI	bp	0/8	5/8	2/a	top
IBL3707	606	VI	bp	0/8	4/8	2/a	top
IBL3693	607	VI	bp	0/8	5/8	2/a	top
IBL3783	608	VI	att am	2/8	4/8	3/b	middle
IBL3766	609	VI	att am	3/8	5/8	3/a	middle
IBL3679	610	UNCLUSTERED	bp	2/8	2/8	1/a	middle

Abbreviations: am-amorphous, att-attached, bp-bipyramidal, cu-cubic, irr-irregular, trap-trapezoidal
